# The potential of molecular markers to improve interventions through the natural history of oesophageal squamous cell carcinoma

**DOI:** 10.1042/BSR20130063

**Published:** 2013-08-14

**Authors:** Nathalia Meireles da Costa, Sheila Coelho Soares Lima, Tatiana de Almeida Simão, Luis Felipe Ribeiro Pinto

**Affiliations:** *Programa de Carcinogênese Molecular, Instituto Nacional de Câncer, Rio de Janeiro/RJ, Brazil; †Departamento de Bioquímica, Universidade do Estado do Rio de Janeiro, Rio de Janeiro/RJ, Brazil

**Keywords:** early diagnosis, oesophageal squamous cell carcinoma, aetiology, molecular biomarkers, prevention, BCL3, B-cell CLL/lymphoma 3, CCRT, concurrent chemoradiation therapy, CRP, C-reactive protein, EAC, EC–adenocarcinoma, EC, oesophageal cancer, EGFR, epidermal growth factor receptor, ESCC, oesophageal squamous cell carcinoma, HER2, human epidermal growth factor receptor 2, HNSCC, head and neck squamous cell carcinoma, HPV, human papillomavirus, IL6, interleukin 6, JAK, Janus kinase, LINE1, long interspersed nucleotide element 1, miRNA, microRNA, SPRR3, small proline-rich protein 3, SPT, second primary tumour, STAT, signal transducer and activator of transcription, TFF1, trefoil factor 1, TP53, tumour protein p53

## Abstract

EC (oesophageal cancer) is one of the ten most frequent and fatal tumours worldwide and ESCC (oesophageal squamous cell carcinoma) accounts for about 80% of the cases. The first symptoms of ESCC arise late during the progression of the disease and, therefore, the diagnosis is usually done in advanced stages. This leads to an inefficient treatment and consequently to a poor prognosis. Thus, a comprehensive knowledge of ESCC biology is of major importance to identify risk factors, especially in high-incidence areas and biomarkers which could enable ESCC prevention and interventions throughout the natural history of the disease. In this review, we present the current knowledge regarding ESCC aetiology as well as the different genetic and epigenetic alterations already described in this tumour. We also discuss how these alterations could be used to anticipate ESCC diagnosis as well as how they can help improving treatment. A molecular natural history of the disease is proposed pointing out potential markers that may improve interventions at different points of ESCC development. Only when the different layers of complexity behind this tumour are elucidated, it will be possible to successfully perform prevention at different levels.

EC (oesophageal cancer) is a highly fatal malignancy and accounts for the sixth most frequent cause of cancer-related deaths worldwide [1,2]. There are two main histopathological types of EC: EAC (oesophageal adenocarcinoma) and ESCC (oesophageal squamous cell carcinoma) that differ considerably in associated aetiological factors and geographical incidence. Despite the increasing incidence of EAC in developed countries in the last decades, ESCC still dominates the EC landscape representing approximately 80% of all cases [3] and is the focus of this review.

The proposed natural history of ESCC is described in Figure 1. It is based on a model commonly used to other diseases [4] and it describes the different stages through which the disease progresses and where intervention may occur. Briefly, after a long-time exposure to aetiological agents, neoplastic transformation occurs in the oesophageal mucosa, which is rarely detected at early stages. When curative therapy is possible, surgery is the primary treatment. For most patients, however, tumour is detected at late stages, when neoadjuvant chemoradiotherapy followed by oesophagectomy may be applied. Unfortunately, most of the patients will not succeed in having a long-time survival. Intervention may occur as primary prevention through the identification of and decreasing exposure to the main aetiological factors, such as tobacco smoking and/or alcohol consumption, whereas secondary prevention may take place through screening programmes. Tertiary prevention may occur through the detection of ESCC at early stages and curative intervention such as oesophagectomy, and quaternary prevention may occur through good quality palliative care. Therefore we will discuss how molecular markers can help to improve the chance of increasing success in each of these possible intervention points.

**Figure 1:**
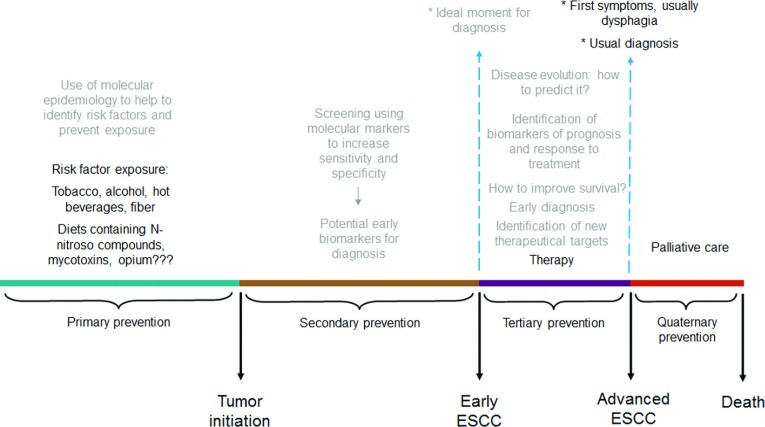
The natural history of ESCC The figure shows the progression of ESCC across time and the different moments for intervention. Bold text describes current knowledge regarding ESCC aetiology, the usual moment of diagnosis and the current therapy modalities. Grey text describes the approaches which could improve diagnosis and treatment and, therefore, improve survival.

So, primary intervention may occur through a better knowledge and identification of the aetiological agents associated with ESCC. However, the aetiology of ESCC is highly complex and may happen through multilayers of exposure to different agents, with different combinations of them affecting different populations, reflected by the remarkably difference in incidence figures observed worldwide, as shown in Figure 2 [1,5]. The region known as ‘Central Asian Oesophageal Cancer Belt’, which stretches from Caspian Sea to Central China, together with southern and eastern Africa and parts of South America, represent the high-incidence risk areas where incidence rates are in the range of 50–150/10^5^ inhabitants [1,2,6]. The very high-incidence areas of Asia and Africa do not have well-established aetiological factors, and differences in incidence rates among men and women are not observed [6–9]. The habit of drinking very hot beverages, diets containing N-nitroso compounds, mycotoxins and lacking antioxidants, and opium consumption are some of the suggested aetiological factors [10,11]. Curiously, in Western countries, where much lower incidence rates are observed, heavy alcohol intake and tobacco consumption are well-established main risk factors for ESCC, and men are 3- to 5-fold more probably to develop ESCC than women, reflecting the higher preponderance of these habits among men [5,7]. Hot mate drinking, is also an independent risk factor present in the highest incidence area in Western World, involving South of Brazil, Uruguay and North of Argentina [10–12], supporting the hypothesis that the presence of a different number of aetiological factors in different populations results in the observed differences in incidence rates.

**Figure 2:**
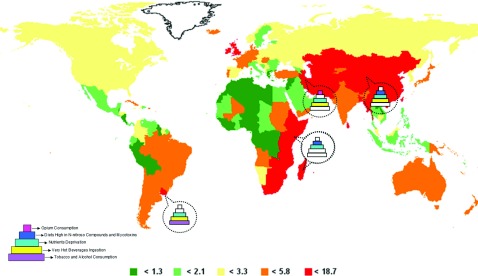
Aetiological agents associated with ESCC in high-risk areas Oesophageal cancer incidence map (created using the incidence/mortality tool on the International Agency for Research on Cancer GLOBOCAN 2008, http://globocan.iarc.fr; accessed in May, 2013) presenting the risk factors associated with ESCC in the different high-incidence areas, which are shown in red, and aetiological factors as a multilayer model representing the exposure to the risk agents, with different combinations of them affecting different populations. The aetiological agents are represented by different colours and white means the absence of the participation of the risk factor in a given area.

Epidemiological studies have demonstrated the association between carcinogen exposure and cancer in human populations. The type, route and amount of exposure can determine the type of cancer. It also influences the type of genetic, genomic and epigenetic alterations, leading in some instances to genetic changes that are ‘signatures’ of specific environmental carcinogens. ‘Carcinogen DNA fingerprints’ arise when a particular type of alteration is frequently observed following exposure to a specific type of carcinogen. Mutations in the tumour suppressor gene *TP53* (tumour protein p53) are the most frequent alterations in ESCC, being present in approximately 45% of the tumours [13] and occur early in the neoplastic progression, since they can be detected also in the normal surrounding oesophageal tissue [14]. About half of all human cancers harbour somatic mutations in *TP53* and more than 15000 mutations leading to the loss of activity of this gene have already been described [15]. This large and diverse spectrum of *TP53* mutations is very helpful in understanding the origin of mutations in human tumours. Therefore *TP53* mutational analysis may help to identify the aetiological factors involved in the onset of ESCC. Studies have reported a prevalence of *TP53* mutations in ESCC varying from 35 to 89%, depending on the geographical region. In ESCC from two medium-incidence areas of the disease (South eastern Brazil and South eastern France), a frequency of about 34% of *TP53* mutations was observed and the mutation profile showed a high percentage of alterations at A:T base pairs [16,17]. This type of mutation can be assigned to the effects of acetaldehyde, the first metabolic product of ethanol [18] and reflects the important role of this risk factor to ESCC in these geographical areas. *TP53* mutation frequency increases consistently in the high-incidence regions reaching rates of over 80% in a high-risk area of France (Normandy) [19], 40–70% in China [20,21], 50–65% in Northern Iran (Tehran) [22,23] and an extremely high prevalence of 90% in North eastern Iran (Golestan province) [24]. The mutation pattern observed in these reports is quite complex and heterogeneous which suggests the diversity of exposures and mechanisms involved in the initiation and/or progression of ESCC in the high-risk areas, making it difficult to point out one specific aetiological factor involved. However, a particular characteristic of *TP53* mutation profile in tumours from high-incidence areas is a large proportion of transitions at CpG sites. Studies conducted in Northern Iran and in Southern Brazil showed a highly representative percentage of 33 and 18%, respectively, of G to A transitions at CpG sites among all mutations detected in the *TP53* gene [22,23,25]. Transitions at CpG sites may be a consequence of spontaneous deamination of 5-methylcytosine to thymine, but it may also be caused by chronic inflammation due to high levels of NO (nitric oxide) produced in the inflammatory process [26,27], being frequently found in cancers occurring within an inflammatory context [28,29]. The ingestion of very hot beverages is a cultural habit in patients with ESCC from high-incidence areas [22,30] and one of the best characterized risk factors associated with its development in these regions [10]. The thermal injury caused by the ingestion of very hot beverages can chronically irritate the oesophageal mucosa generating an inflammatory process, and also diminishes the barrier function of the epithelium against exposure to carcinogens [31,32]. Further supporting the notion that the inflammatory context caused by the consumption of beverages at high temperatures in the high-risk areas may account to the large proportion of transitions found at CpG sites of *TP53*, recently, a study performed in Golestan (Iran) observed that G to A transition, especially at CpG sites, occurs most frequently in ESCC from patients who drink tea at the highest temperatures [24].

HPV (human papillomavirus) infection has been suggested to have a role in ESCC [33] and controversial results have arisen, probably due to low number of cases, variations in the sensitivity of detection techniques, methodologies for processing samples and study design employed [34]. However, a recent report using larger number of patients and more robust methodologies, including the use of surrogate markers such as *TP53* mutations and CKI (cyclin-dependent kinase inhibitor) 2A (p16) overexpression, have shown that HPV presents a minor role, if any, in ESCC development [35–37]. Furthermore, differently from oropharyngeal tumours [38], patients with HPV positive ESCCs do not present a better prognosis when compared with patients with HPV negative tumours [37].

The 5-year overall survival for ESCC dramatically falls when tumours are not detected at early stages (Stages IA and IB), demonstrating the need for early ESCC detection through screening programmes (secondary prevention) [39]. The squamous epithelium dysplasia is recognized as a premalignant lesion, but usually it is not observed during simple endoscopy. After introduction of Lugol chromoendoscopy (Schiller's test), the observation of unstained dysplasic areas in this epithelium became possible [40,41]. However, chromoendoscopy is an expensive method, requiring specialized equipment and trained professionals, and the common presence of Lugol-negative staining in inflammatory areas along the oesophageal mucosa may compromise sensitivity and specificity [42], hampering the performance of this screening procedure.

With the purpose of early detection of ESCC in areas of high incidence, a technique for collecting oesophageal cytology material with balloon or sponge samplers was developed in China in the end of the 1950s. These samplers are introduced into the stomach, inflated and then withdrawn through the oesophagus, scraping and collecting exfoliated oesophageal cells [40]. Although this technique is easy to perform and well tolerated by patients, the cytological analysis of the collected material showed low sensitivity when compared with histopathological analysis, and also great inter- and intra-observer variation [43,44].

Recently, several molecular markers were identified for predicting ESCC, such as the SPRR3 (small proline-rich protein 3). Its expression is highly induced during the differentiation of human epidermal keratinocytes and has been considered a differentiation marker for squamous epithelium [45,46]. This is abundantly expressed in oral and oesophageal epithelium and the alterations in *SPRR3* mRNA and/or protein expression already observed in surrounding mucosa may represent an early molecular event related to the field cancerization [46–48].

The ‘field cancerization’ phenomenon was first described by Slaughter et al. in 1953 [49]. They examined histological slides of HNSCC (head and neck squamous cell carcinoma) in an attempt to understand the changes found in the surrounding tissue of these tumours to try to explain why these patients develop multiple SPTs (second primary tumours) along the squamous epithelium of the upper aerodigestive tract. Microsatellite-based analysis of LOH (loss of heretozygosity) and *TP53* mutation status has been studied in patients with HNSCC and SPT in order to understand which mechanisms are involved in this process [50,51]. Braakhuis et al. [52] proposed a classification of SPT in larynx, lung or oesophagus based on the molecular profile of the tumours: it is assigned metastasis when the molecular alterations are similar; it is assigned second field tumours, derived from the same genetically altered field as the primary tumour, and SPT when the molecular profiles of both tumours are different.

Besides genetic alterations, epigenetic changes have also been described as important contributors during ESCC development. Specifically, epigenetic alterations seem to occur early during this process and, therefore, have emerged as potential diagnostic biomarkers for ESCC. Several studies have focused on the role of differential methylation in the promoter region of individual genes on ESCC development. It has been shown that the promoter of the gene that encodes the LMP7 (low molecular-mass protein 7) is hypermethylated in tumours when compared with neighbouring normal tissues [53]. However, this was not the first time epigenetic alterations were pointed out as early changes. A previous study from our group, based on microarray analysis, showed that the methylation status of *BCL3* (B-cell CLL/lymphoma 3), a member of the nuclear factor of κ light polypeptide gene enhancer in B-cells inhibitors [IκB (inhibitory κB)] family, and the *TFF1* (trefoil factor 1), a protector of the mucosa, are disrupted in ESCC and in the normal surrounding tissue [54]. In this case, the *TP53* mutation profile had been previously analysed in the same set of samples and no mutations were found in the normal adjacent mucosa [17], evidencing that epigenetic alterations may occur even before the supposed first genetic alterations. Another important genomic feature often found hypomethylated in tumours are the repetitive elements. Different studies have already shown the hypomethylation of the LINE1 (long interspersed nucleotide element 1) in ESCC [54–57] and although this methylation profile was not correlated with tumour staging [57], Iwagami et al. [56] showed a correlation with prognosis. Besides LINE1, the methylation status of Alu repeats was also investigated in ESCC [55]. In this study, not only the tumours showed lower methylation levels, but also their normal counterparts, in comparison with the oesophageal mucosa from healthy individuals. These results suggest, therefore that Alu hypomethylation could be an early change during oesophageal carcinogenesis and contribute to the cancerization field. Deregulation of miRNA (microRNA) also seems to be involved in every step of ESCC development. Yang et al. [58] have recently shown that miR-338-3p, miR-218 and miR-139-5p are down-regulated in ESCC compared with the adjacent non-tumour tissues, whereas miR-183, miR-574-5p, miR-21 and miR-601 are up-regulated. Multiple regression analysis revealed that the aberrant expression of miR-338-3p, miR-139-5p, miR-574-5p and miR-601 increased the risk of EC. Furthermore, miR-21 was significantly increased in heavy drinking patients. Therefore there is a set of differentially expressed miRNAs in EC that may be associated with the incidence and development of ESCC and even with risk factor exposure. Furthermore, different miRNAs have been shown to predict prognosis in ESCC, such as miR-375, miR-192, miR-21, miR-181b, miR-146b and miR-150, among others [59,60]. This is of special interest because miRNAs can be easily detected in the serum, since they are secreted by various cells in exosomes. Therefore they may represent a non-invasive way to predict prognosis in ESCC. Tanaka et al. [61] have investigated the exosomal levels of miR-21 in patients with ESCC and they found that its expression is significantly higher in this group than in patients with benign diseases with and without systemic inflammation [CRP (C-reactive protein) <0.3 mg/dl]. Furthermore, miR-21 was not detected in the serum that remained after exosome extraction and exosomal miR-21 expression was correlated with advanced tumour classification, positive lymph node status and the presence of metastasis with inflammation or/and clinical stage without inflammation (CRP <0.3 mg/dl). This might be one of the first steps towards the utilization of epigenetic markers in ESCC diagnosis and prognosis determination. However, in parallel, efforts must be made to improve ESCC treatment.

ESCC therapy is defined according to the primary tumour dimensions and its locoregional lymph nodes involvement [62]. Surgery is considered the gold standard treatment for ESCC and consists on near total oesophagectomy with cervical, thoracic and abdominal lymphadenectomy. However, prognosis of patients with EC remains extremely poor even after this radical surgery is performed. Poor response to curative oesophagectomy is mainly explained by early ESCC systemic dissemination due to the morphological characteristics of this tissue, since it presents a rich lymphatic drainage in the submucosal layer which allows micrometastasis even in early stages tumours [63].

Thus, owing to the low effectiveness in curative oesophagectomy, other therapeutic modalities have been studied as combined treatment to surgical resection, such as adjuvant and neoadjuvant chemoradiotherapy. It has been reported that the accepted standard of care is neoadjuvant chemoradiotherapy followed by surgical resection, although this treatment modality remains controversial [64]. Nevertheless, two recent studies have shown a significant increase in overall survival among patients with ESCC treated with chemoradiation with paclitaxel and cisplatin [65] or carboplatin [66].

Several molecular markers have already been reported to predict response to treatment in ESCC. One of the best candidates is *TP53*. Okumura et al. [67] have reviewed this issue and showed that, whenever a correlation was found, a negative immunohistochemical staining for p53 could predict the group of responders to neoadjuvant chemoradiotherapy. In addition, a meta-analysis with 28 included studies showed that the wild-type p53 status (low expression of p53 protein and/or wild-type *TP53* gene) is associated with high response to chemotherapy-based treatment in EC [68]. These reports clearly show the relevance of *TP53* to treatment response in ESCC, although this marker is not currently employed in the clinics.

In the attempt to find a wider panel of biomarkers for treatment response in ESCC, a recent study has evaluated the expression level of 16 proteins, including p53, in ESCC treated with CCRT (concurrent chemoradiation therapy) [69]. Although the number of patients included in the study was quite small, the authors showed that high levels of MIB1 (mindbomb E3 ubiquitin protein ligase 1) and low levels of nuclear factor of kappa light polypeptide gene enhancer in B-cells [NF-κB (nuclear factor κB)], HER2 (human epidermal growth factor receptor 2) and ER (oestrogen receptor) are good prognostic factors following definitive CCRT for ESCC.

Besides genetic markers, epigenetic alterations have also been shown to predict response to treatment. Sugimura et al. [70] have evaluated the expression profile of 365 miRNAs in cisplatin-resistant cells and found a signature of 15 miRNAs that could be involved in this resistance phenotype. This signature was then evaluated in pretreatment biopsy samples from 98 patients with EC who received preoperative chemotherapy. Interestingly, low expression of let-7b and let-7c in before-treatment biopsies from 74 patients of the training set correlated significantly with poor response to chemotherapy, both clinically and histopathologically. This relationship was further confirmed in the other 24 patients of the validation set. The authors also showed that the resistance to cisplatin is mediated, at least in part, by IL6 (interleukin 6) since EC cells release this cytokine after exposure to cisplatin and IL6 activates prosurvival JAK/STAT3 (Janus kinase/signal transducer and activator of transcription 3) pathway in an autocrine manner. In this context, transfection of let-7c was shown to restore sensitivity to cisplatin and increase rate of apoptosis by directly repressing cisplatin-activated IL6 (interleukin 6)/STAT3 prosurvival pathway [70].

In the same context, Chen et al. [71] have evaluated the potential of IL6 to be used as a biomarker of treatment response in ESCC. Among the 173 EC tissues analysed, 88 (51%) showed positive IL6 immunoreactivity, and there was a positive correlation between IL6 overexpression and tumours developing locoregional or distant metastasis. Furthermore, the expression of IL6 was significantly associated with a lower rate of pathological response to curative treatment. Regarding treatment, the authors also showed that IL6 silencing significantly sensitized EC cells to irradiation *in vitro*. Finally, serum IL6 levels were significantly elevated in patients with locoregional or distant metastasis compared with those in patients with disease control [71]. All the data presented here suggest an important role for IL6 signalling in ESCC establishment and progression and reveals IL6 potential not only as a predictor of treatment response but also as a new target therapy. Finding new targets for therapy in ESCC should be a priority because, although all the recent advances in identifying biomarkers for treatment response, the group of non-responders to the conventional therapy still does not have any alternative option of treatment.

Once it has been shown that IL6/JAK/STAT3 prosurvival pathway is important in ESCC progression [70,71], an interesting alternative for ESCC treatment may emerge from these findings. Recently, a new group of drugs, the JAK inhibitors, was developed and is being successfully used to treat myelofibrosis [72]. Furthermore, overactivation of the JAK/STAT pathway with or without JAK protein mutations has been reported in subsets of patients with certain solid tumours and haematologic malignancies and some clinical trials using JAK inhibitors are already ongoing for acute leukaemia, pancreatic and breast cancer [72].

Another possible target for ESCC treatment has emerged from observations in HNSCC. The EGFR (epidermal growth factor receptor) is overexpressed in a high percentage of the cases (90%) and, therefore these patients were considered good candidates to benefit from EGFR-targeted therapies [73]. Although this strategy has shown some success in HNSCC, its potential to treat ESCC had been slightly accessed. In this context, our group has recently published a report showing that EGFR and HER2 overexpression are not common events in ESCC, nor are mutations in *EGFR*, v-Ki-ras2 Kirsten rat sarcoma viral oncogene homologue (*KRAS*) or v-raf murine sarcoma viral oncogene homolog B1 (*BRAF*) [74]. Therefore most patients with ESCC do not have the molecular profile for anti-HER targeted therapy, which shows that other markers should be investigated.

Finally, a very exciting new class of drugs has emerged, the epigenetic drugs. So far, such class includes DNMTi (inhibitor of DNA methyl-transferases) and HDACi (inhibitor of histone deacetylases) of which some are already in clinical trials for different types of tumour [75]. These drugs are being seen with great enthusiasm because, for the first time, it is possible to aim the reactivation of a tumour suppressor gene. Epigenetic silencing of tumour suppressor genes is a common event in different malignancies, either by DNA hypermethylation and/or histone modifications and, as epigenetic alterations are reversible, it might be feasible to reverse such states and recover the normal gene expression profile. Although this may sound as a distant future, patients with myelodysplastic syndrome are already profiting from these new treatment modalities and soon this might also be true for patients with cancer, including those diagnosed with ESCC.

So, Figure 3 proposes a view of the natural history of ESCC with the perspective of the potential use of molecular markers. It is not a broad view of all of the potential molecular markers revealed in different studies, but a concise one, based on the discussion presented in this review (Table 1). Molecular epidemiology markers, such as *TP53* mutation profile can help to better identify aetiological agents and mechanisms related to early neoplastic transformation of the oesophageal mucosa. Epigenetic events that contribute to the cancerization field of the oesophageal mucosa, such as differential methylation of *TFF1* and *BCL3* may be used to assess early malignant transformation, whereas *SPRR3* down-regulation and alteration in the expression of miRNAs offer the possibility to better identify early intramucosal tumours. Different markers present not only in the tumour cells, but also in the tumour microenvironment and released in blood such as IL6, not only present the potential to predict prognosis, but also offer new potential target therapies to improve therapy efficacy. Many of these markers have to be tested and validated in specific clinical studies before they may be incorporated into clinical practice. However, considering the potential to improve interventions throughout the natural history of ESCC, the incorporation of molecular markers into the clinical routine seems to be the best horizon to improve patients with ESCC outcome.

**Table 1:** Potential molecular markers that may contribute to the different levels of prevention in ESCC

Gene	Function	Alteration in ESCC	References
Molecular biomarkers for primary prevention			
* TP53*	Tumour suppressor gene	Mutations	[16,17,19–24]
* BCL3*	Proto-oncogene candidate	Promoter hypomehylation	[54]
* TFF1*	May protect the mucosa from insults	Promoter hypermethylation	[54]
Alu elements	Retrotransposon	Hypomethylation	[55]
Molecular biomarkers for secondary prevention			
* SPRR3*	Differentiation marker of squamous epithelium	Down-regulation	[46–48]
miR-21	miRNA with 382 predicted targets*	Up-regulation	[58]
miR-139-5p	miRNA with 349 predicted targets*	Down-regulation	[58]
miR-183	miRNA with 276 predicted targets*	Up-regulation	[58]
miR-218	miRNA with 509 predicted targets*	Down-regulation	[58]
miR-338-3p	miRNA with 295 predicted targets*	Down-regulation	[58]
miR-574-5p	miRNA with 169 predicted targets*	Up-regulation	[58]
miR-601	miRNA with 104 predicted targets*	Up-regulation	[58]
Molecular biomarkers for tertiary and quaternary prevention			
* TP53*	Tumour suppressor gene	Mutations	[67,68]
* IL6*	Cytokine involved in inflammation	Up-regulation	[70,71]
* LINE1*	Retrotransposon	Hypomethylation	[56]
miR-21	miRNA with 382 predicted targets*	Up-regulation	[60]
miR-146b	miRNA with 243 predicted targets*	Up-regulation	[60]
miR-181b	miRNA with 1080 predicted targets*	Up-regulation	[60]
miR-375	miRNA with 30 predicted targets*	Down-regulation	[59]
let-7b	miRNA with 208 predicted targets*	Down-regulation	[70]
let-7c	miRNA with 210 predicted targets*	Down-regulation	[70]

*Target prediction according to miRDB database.

**Figure 3:**
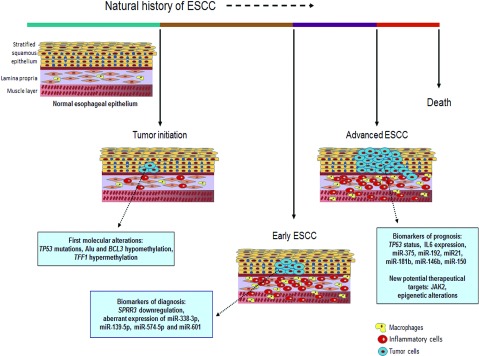
The molecular natural history of ESCC The figure shows the progression of ESCC once the normal oesophageal epithelium is exposed to risk factors. Tumour initiation is characterized by the arising of the first transformed cells, which carry the first molecular alterations, such as *TP53* mutations and aberrant DNA methylation. As the tumour progresses, the crosstalk between tumour and inflammatory cells gain importance for supporting the growth of the neoplastic mass. This is the ideal moment for diagnosis since tumour is usually confined to the mucosa and did not invade other tissues. With this purpose, molecular alterations exclusively found in tumour cells have been identified, such as SPRR3 down-regulation and miRNA deregulation, and may help to achieve an early diagnosis. If no intervention is made, tumour usually progresses to an invasive form and, at this point, the prognosis of the patients diagnosed with ESCC is rather poor, once treatment is not effective. Therefore it is of major importance to identify biomarkers that can predict prognosis and identify the most suitable treatment for each case. Furthermore, new potential therapeutical targets emerge as a promise to improve survival.
